# Comparing the Effectiveness of Smartphone Applications in the Measurement of Interpupillary Distance

**DOI:** 10.7759/cureus.42744

**Published:** 2023-07-31

**Authors:** Kenneth D Han, Muhammed Jaafar, Isabella M Stoakes, Phillip C Hoopes, Majid Moshirfar

**Affiliations:** 1 Medicine, University of Arizona College of Medicine-Phoenix, Phoenix, USA; 2 Osteopathic Medicine, Pacific Northwest University of Health Science, Yakima, USA; 3 Ophthalmology, Hoopes Vision, Draper, USA; 4 Corneal and Refractive Surgery, HDR Vision Research Center, Hoopes Vision, Draper, USA; 5 Department of Ophthalmology and Visual Sciences, John A. Moran Eye Center, University of Utah School of Medicine, Salt Lake City, USA; 6 Corneal Transplantation and Eye Banking, Utah Lions Eye Bank, Murray, USA

**Keywords:** warby parker, eye measure, pdcheck ar, digital pupilometer, pd self-measurement, ordering glasses online, pupilometer, pd, ipd, pupillary distance

## Abstract

Purpose

To determine the accuracy of three smartphone applications in the measurement of interpupillary distance (IPD).

Methods

This study compared measurements from three smartphone applications to measurements obtained by a single trained examiner using a digital pupilometer in 44 subjects. The mean absolute error (MAE) of IPD prediction by each application was compared. Additionally, the frequency at which each application measured IPD within ± 0.05 mm, ± 0.10 mm, ± 0.25 mm, ± 0.50 mm, ± 0.75 mm, and ± 1.00 mm of the digital pupilometer measurement was determined.

Results

The Eye Measure (Dotty Digital, Sydney, New South Wales, Australia) and Warby Parker (Warby Parker, New York, New York) applications had significantly lower MAE of IPD measurements (0.511364 mm) compared to the PDCheck AR (EyeQue Corp., Newark, California) application (1.375 mm). The Warby Parker application most frequently obtained accurate IPD measurements within the following ranges: ± 0.05 mm, ± 0.10 mm, ± 0.25 mm, ± 0.50 mm, ± 0.75 mm, and ± 1.00 mm.

Conclusion

Of the three smartphone applications compared in this study, the Warby Parker application performed to the highest degree of accuracy and may serve as an adequate alternative when conventional IPD measurement methods are either unavailable or unable to be performed accurately.

## Introduction

The interpupillary distance (IPD), also known as the pupillary distance, is the distance between the centers of the pupillary light reflexes of both eyes [1]. Obtaining an accurate IPD measurement is crucial, as it allows for proper alignment of the visual axis of the eye with the optical center of the lens. This parameter can also be used as a clinical landmark to aid in the identification of impairments in accommodation and near-point convergence [2], as well as other vision issues [3]. Furthermore, IPD can be utilized in the identification of craniofacial abnormalities such as craniofacial dysostosis (Crouzon’s disease), frontonasal dysplasia (median cleft face syndrome), acrocephalosyndactyly (Apert’s syndrome), and fetal hydantoin syndrome [4]. This measurement is traditionally obtained through the use of a pupilometer or a pupillary distance ruler and should be accurate within 0.50 mm [5].

IPD measurements performed by untrained patients on themselves or others are inaccurate and unreliable [6]. This may have ramifications in the setting of ordering glasses online, as patients often provide eyeglass companies with self-measured IPD values, which may result in improper lens centering and vision correction.

Recently, new smartphone applications such as PDCheck AR (EyeQue Corp., Newark, California), Eye Measure (Dotty Digital, Sydney, New South Wales, Australia), and the Warby Parker (Warby Parker, New York, New York) application have emerged as potential solutions to this problem. These applications are quick and easy to use, taking no more than 10-30 seconds to obtain an IPD measurement.

While previous studies have examined the efficacy of smartphone applications in measuring other ocular parameters [7,8], to the best of our knowledge, no study has been conducted analyzing the accuracy of currently available smartphone applications in the measurement of IPD. Therefore, the goal of this article is to determine the accuracy of three different smartphone IPD-measuring applications in comparison to a medical-grade pupilometer to determine if these applications can serve as reliable alternatives to obtaining IPD measurements.

## Materials and methods

This is an analysis of the PDCheck AR, Eye Measure, and Warby Parker smartphone applications compared to a digital pupilometer (Model 9AT, Huanyu High-Tech Co., Ltd., Wenzhou Bridge Industrial Park, China). All measurements were performed by a single trained examiner in June 2023 at Hoopes Vision in Draper, Utah. Forty-four subjects aged 20 to 75 years old (31.8% male, 68.2% female) were included in this study. Any subjects with facial deformities or other structural ocular pathology were excluded. Informed consent was obtained from all subjects to participate in this study. This study was approved by the Hoopes Vision Ethics Review Board and adheres to the Declaration of Helsinki. 

The authors searched the Apple iPhone store using keywords such as “pupillary distance,” “interpupillary distance,” and “PD.” The following applications were selected as they would be the three top applications that any Apple iPhone store user would find when trying to determine their IPD measurement: PDCheck AR, Eye Measure, and the Warby Parker application. The authors have no affiliation with any of these applications.

Binocular distance IPD was measured using four methods in the following order for each patient: a digital pupilometer, PDCheck AR, Eye Measure, and the Warby Parker application. The digital pupilometer was placed on the nose bridge of each subject, and the manufacturer’s instructions were followed for each measurement. The digital pupilometer measurements performed by the examiner were considered the gold standard for this study. Instructions for use were followed for each application, respectively.

Data collection and analysis were performed in Microsoft Excel (Microsoft, Redmond, Washington, USA) and IBM SPSS Statistics for Macintosh, Version 28.0., and G*Power (version 3.1, Franz Faul, Universitӓt Kiel, Germany). The Shapiro-Wilk test was used to determine the normality of the data distribution. With a medium effect size (0.5) and an alpha level of 0.05, the minimum sample size to achieve a statistical power of 0.80 was 35. Post hoc analysis with the study sample size of 44 revealed a statistical power of 0.89.

The mean absolute error (MAE) of the IPD measurement from each smartphone application compared to the digital pupilometer IPD measurement was calculated for each participant. After determining that our data were non-normally distributed, the Wilcoxon Signed-Rank test was used to compare the MAE of each smartphone application’s IPD measurement. In addition, the percentage of eyes with MAE of IPD measurement within 0.05 mm, 0.10 mm, 0.25 mm, 0.50 mm, 0.75 mm, and 1.00 mm from the pupilometer measurements for each application were also calculated. The Bonferroni correction was used to adjust the alpha level to 0.02 (rounded up from 0.0167).

## Results

IPD ranged from 55.5 mm to 71 mm, with the mean IPD being 61.8 mm (Table 1).

**Table 1:** Population Demographics and Interpupillary Distance Measurements IPD = Interpupillary Distance

Demographics	
Number of Subjects	44
Average Age (years)	38 ± 14.4
Male (%)	31.8
Female (%)	68.2
Caucasian (%)	81.8
East Asian (%)	4.55
Hispanic (%)	4.55
South Asian (%)	4.55
Middle Eastern (%)	4.55
Average IPD (mm)	61.8 ± 3.6

**Figure 1:**
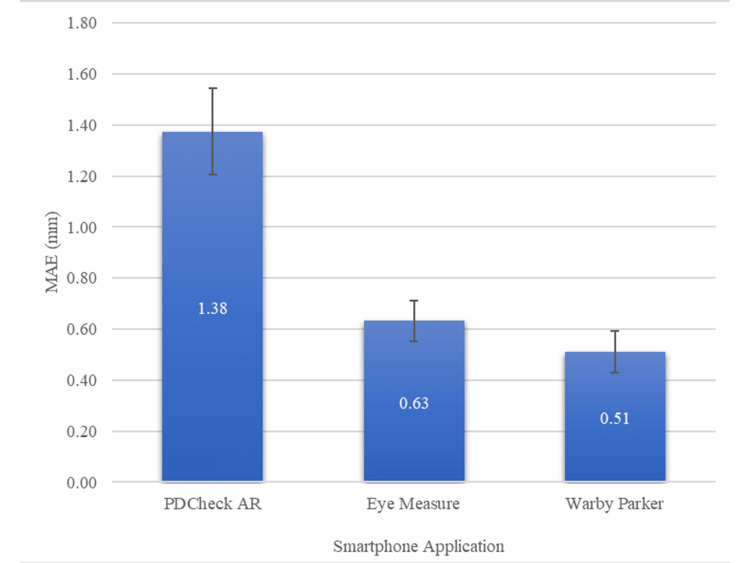
The MAE of IPD for each application (PD Check AR, Eye Measure, and Warby Parker) from the pupilometer interpupillary distance. MAE = mean absolute error, IPD = interpupillary distance, PD = pupillary distance, AR = augmented reality, *,** = Statistical significance

When compared to the IPD measurement from the digital pupilometer, the PDCheck AR application was the least accurate, having a significantly higher MAE compared to the Eye Measure (P < 0.001) and Warby Parker (P < 0.001) applications. However, there was no statistically significant difference between the Warby Parker and Eye Measure MAE values. (P = 0.192) (Figure 1).

**Figure 2:**
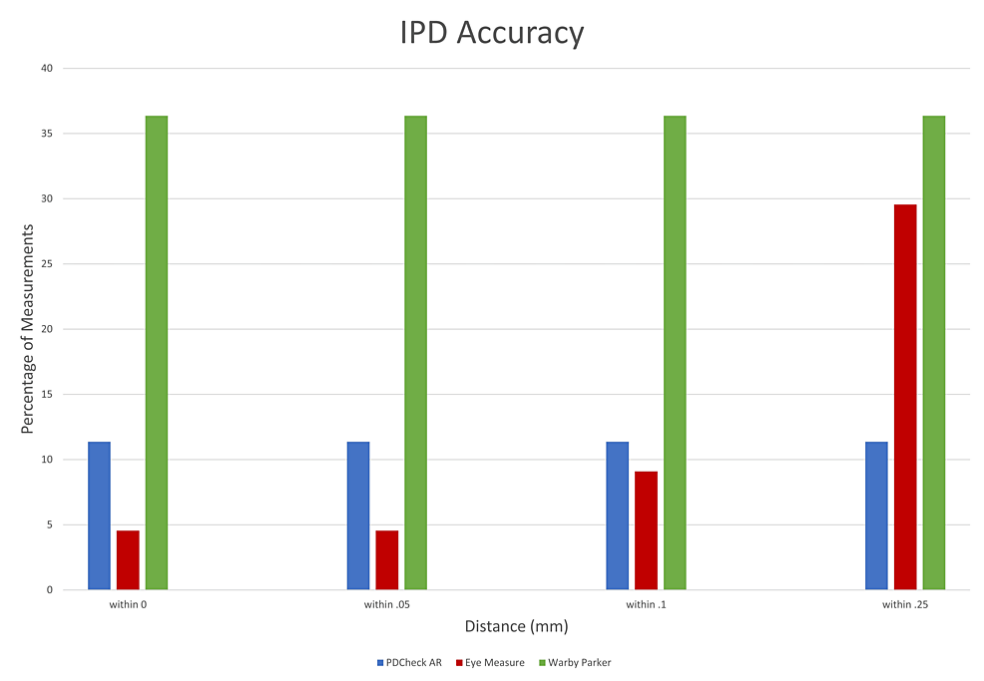
The percentage of interpupillary distance measurements of the three applications (PD Check AR, Eye Measure, and Warby Parker) within +/- 0 mm, +/- 0.5 mm, +/- 0.1 mm, and +/- 0.25 mm from the pupilometer measurement. IPD = interpupillary distance, PD = pupillary distance, and AR = augmented reality

Additionally, the Warby Parker application had the highest percentage of IPD measurements within ± 0.05 mm, ± 0.10 mm, ± 0.25 mm, ± 0.50 mm, ± 0.75 mm, and ± 1.00 mm of the digital pupilometer measurements, outperforming both the PDCheck AR and Eye Measure applications (Figure 2).

## Discussion

Accurate IPD measurement is an essential factor in the prescription of eyeglasses, as it allows clinicians to properly align the optical center of each lens with the visual axis of a patient’s eyes [9]. These findings are especially pertinent given the recent rise in individuals ordering prescription eyeglasses from online retailers. Gordon et al. demonstrated that nearly one out of 10 eyeglasses ordered online failed to meet the national optical quality standards [10]. Given the fact that eyeglass prescriptions often lack IPD measurements, it is conceivable that erroneous IPD measurements account for a significant proportion of these prescription errors [11]. Thus, accessible, easy-to-use tools for measuring IPD must be available to the public since many do not have the luxury of being able to re-purchase eyeglasses after receiving an incorrect pair. The main goal of this study was to determine the accuracy of the IPD measurement of three smartphone applications compared to the use of a digital pupilometer by a trained examiner.

Of the three highest-rated and free IPD measurement applications identified by the authors from the Apple iPhone store, the Warby Parker application predicted IPD with the highest degree of accuracy. This application achieved the lowest MAE of IPD measurement and most frequently measured IPD within 0.10 mm, 0.25 mm, 0.50 mm, 0.75 mm, and 1.00 mm when compared to the digital pupilometer. The authors of this study speculate that these findings are attributed to the more organized, stepwise instructions provided by the application during IPD measurement. For example, the application asks the patient to hold their smartphone at arm’s length away and to follow an animated circle around the smartphone screen with their eyes. Additionally, the Warby Parker application prompts patients to make adjustments, such as removing their glasses or tilting their heads in a certain direction, to obtain more accurate measurements. On the other hand, while the PDCheck AR application does have the ability to prompt patients to move their smartphone closer or farther from their face, it does not specify a certain distance at which the smartphone should be held and simply asks patients to look at the center of their smartphone screen. Similarly, the EyeMeasure application does not provide any user instructions.

Although the tests performed in this study achieved statistical power levels greater than the generally accepted 0.80, the sample size was still a potential limitation, and future studies should repeat this analysis with a greater number of subjects. Additionally, while the use of a single trained examiner eliminated inter-rater biases, the extent to which intra-rater bias impacts our results cannot be determined, and future studies should address this potential source of error [12].

## Conclusions

In conclusion, the Warby Parker iPhone application was found to be the most accurate in measuring IPD. This application provided clear instructions to the user and consistently produced IPD measurements closest to those of the digital pupilometer. When patients are unable to receive IPD measurements from trained clinicians, this smartphone application may serve as an acceptable alternative. Future studies should continue exploring affordable, accessible options that can be utilized by untrained patients to improve the quality of eyeglass prescriptions ordered online.
